# Alphabet learning and the uppercase facilitation effect

**DOI:** 10.1007/s11881-025-00360-z

**Published:** 2026-01-09

**Authors:** Somin Park, Shayne B. Piasta

**Affiliations:** 1 Faculty of Education, University of Hong Kong, Hong Kong, China; 2 Department of Teaching and Learning, The Ohio State University, Columbus, United States; 3 Crane Center for Early Childhood Research and Policy, The Ohio State University, Columbus, United States

**Keywords:** Uppercase familiarity effect, Alphabet learning, Letter name learning, Early literacy skills

## Abstract

Alphabet knowledge is a vital learning target during early childhood, including for children who are at risk for reading difficulties. For these children, we need to provide effective, efficient, evidence-based instruction. In this study, we examined the uppercase facilitation effect (i.e., children are more likely to identify lowercase letters when they know the corresponding uppercase forms) in the context of alphabet learning and thus its potential to be leveraged as an instructional strategy. Thirty-six 3:5 to 5:5 years old children at risk for reading difficulties due to low alphabet knowledge (18 girls; 50 months old on average; 22 English monolinguals and 14 emergent bilinguals) participated in a within-subjects experiment. Children received 1:1 alphabet instruction on individually selected letters, with other letters serving as controls. We evaluated participating children’s letter name and sound production pre- and post-instruction. We accounted for letters being nested within each child using multilevel logistic regression. Children were more likely to know lowercase letters if they knew the corresponding uppercase letters, regardless of whether the letters were taught or not taught. The uppercase facilitation effect did not apply in the context of alphabet learning. We discuss instructional implications.

## Alphabet learning and the uppercase facilitation effect

Alphabet knowledge (i.e., the ability to recognize and produce letter names, sounds, and forms) plays a critical role in the literacy achievement of children learning alphabetic orthographies (Georgiou et al., 2012; National Early Literacy Panel, 2008). Theories suggest that alphabet knowledge is closely related to decoding and spelling skills through the process of understanding the alphabetic principle (Byrne & Fielding-Barnsley, 1989; Ehri, 2015) and applying knowledge of letters and sounds to reading and writing (Clayton et al., 2020; Ehri, 2020). Moreover, substantial research has highlighted the association between children’s alphabet knowledge and later reading and spelling (Georgiou et al., 2012; Hjetland et al., 2020; National Early Literacy Panel, 2008; Piasta et al., 2024). The alphabet is therefore an important learning target during early childhood, especially because low alphabet knowledge has been identified as a significant risk factor for reading difficulties, with children who struggle to master letter knowledge more likely to experience later reading challenges (Gallagher et al., 2000; Piasta et al., 2012; Torppa et al., 2006).

One well-known aspect of alphabet knowledge is the uppercase facilitation effect. Studies have shown that children are more likely to identify lowercase letters when they know the corresponding uppercase form (i.e., are able to identify names/sounds for both cases at a given point in time; e.g., Evans et al., 2006; Huang & Invernizzi, 2014; Pence-Turnbull et al., 2010; Treiman & Kessler, 2004). However, all existing studies have been correlational and considered data collected concurrently, such that it is unclear whether the uppercase facilitation effect might be leveraged to provide evidence-based instruction. The effect has not been tested in longitudinal designs that focus on learning over time (i.e., whether children who are initially unable to identify lowercase letter names/sounds can do so at a later point in time). Thus, we investigated whether knowing uppercase letters moderated the impact of alphabet instruction in a sample of children who could be considered at-risk for reading difficulties due to low alphabet knowledge. In testing this effect via a within-subjects experimental design, we provide evidence as to whether uppercase letter knowledge may affect lowercase letter learning and thereby serve as a mechanism underlying children’s alphabet development. In doing so, we consider instructional implications as to whether uppercase letter knowledge can be leveraged as a resource for teaching lowercase letter knowledge for children at risk of reading difficulties.

### Children’s knowledge about the alphabet

Differences in letter and child characteristics contribute to what children know about the alphabet (Kim & Petscher, 2013; Kim et al., 2020; Piasta, 2014). Certain letters are less or more likely to be known by children at given timepoints (Drouin et al., 2012; Piasta et al., 2016). For example, children experience some letters more frequently in print (Kim et al., 2020; Pence-Turnbull et al., 2010) and may practice the first half of the ABC song more often than the second half (e.g., Justice et al., 2006). Some letter names cue their corresponding letter sounds (i.e., acrophonic principle; Piasta, 2014). Moreover, young children in North America tend to be less knowledgeable about lowercase forms compared to uppercase forms (Evans et al., 2006; Pence-Turnbull et al., 2010). Differences also derive from child characteristics. For instance, children tend to identify letters more readily when letters are included in their first name and, in particular, when the letter is the first of their first name (e.g., Justice et al., 2006; Piasta et al., 2016; Treiman et al., 2012; Pence-Turnbull et al., 2010). Furthermore, children’s phonological awareness predicts their letter sound knowledge and learning (Cardoso-Martins et al., 2011; Lerner & Lonigan, 2016; Savage & Carless, 2004); as such, children at risk for reading difficulties due to phonological processing difficulties may experience challenges with letter-sound learning (Melby-Lervåg et al., 2012; Torppa et al., 2010). Moreover, children with a familial risk for dyslexia or reading difficulties often exhibit lower alphabet knowledge, which can further contribute to their reading challenges (Gallagher et al., 2000).

Children’s experiences such as home literacy environments and classroom instructional quality also predict alphabet knowledge as children within the same classroom often exhibit a wide range in alphabet knowledge (see Piasta, 2014; Piasta et al., 2022; Piasta & Wagner, 2010b). Environmental print, such as signs, logos, and product labels, also informs children’s print, letter, and word recognition (Masonheimer et al., 1984; Neumann et al., 2009). Environmental print often features uppercase letters (e.g., STOP signs, brand logos). Moreover, although children’s books primarily present letters in lowercase within continuous text (Fears & Lockman, 2020), adults often emphasize uppercase letters (Gerde et al., 2019). These differences in case forms may also influence children’s familiarity with letters and contribute to the observed uppercase advantage in early alphabet knowledge.

### Alphabet learning and instruction

Alphabet learning is an important instructional target during early childhood for both children at-risk for reading difficulties and their peers (National Early Literacy Panel, 2008; Piasta et al., 2012). In the U.S. as well as other English-speaking countries, national reports have emphasized alphabet knowledge as one of the “key early literacy and reading indicators” (National Early Literacy Panel, 2008, p. 60; see also Rose, 2006; Rowe, 2005), and much attention in early childhood education has focused on teaching code-based skills including alphabet knowledge (Hadley et al., 2024; Pearson & Hiebert, 2010). However, children at risk for reading difficulties often enter first grade with limited alphabet knowledge, struggling to identify letters by name or sound (Sunde et al., 2020; Torppa et al., 2006). Research suggests that children who know fewer than 18 uppercase and 15 lowercase letters by 60 months are more likely to be later identified as at risk on reading measures (Piasta et al., 2012; U.S. Department of Health and Human Services, 2015). Yet, Heilmann et al. (2018) reported that on average, many U.S. children participating in a Head Start program identified only about 11 uppercase letter names by spring, well below the benchmark associated with increased risk of later reading difficulties. Similarly, previous studies have shown that U.S. emergent bilingual children identified 9 to 13 letters on average at the beginning of kindergarten (Cirino et al., 2007; Ford et al., 2013). Challenges that children experience in learning the alphabet require attention because this may lead to reading difficulties later in their academic careers (Piasta et al., 2012, 2024; Torppa et al., 2006).

However, limited evidence exists as to how best to support children’s alphabet learning (National Early Literacy Panel, 2008; Piasta, 2014, 2023; Piasta & Wagner, 2010a). In their meta-analysis Piasta and Wagner (2010a) found only a handful of studies that specifically targeted alphabet knowledge and reported that multicomponent instruction improved children’s alphabet knowledge alongside other literacy skills. Although limited, studies have established that children benefit from explicit instruction in letter names and letter sounds (Piasta et al., 2010, 2022; Park et al., 2025; Roberts et al., 2018), paired-associated learning (i.e., explicitly teaching children by showing them a letter while saying the letter name and sound; Roberts et al., 2018), and embedded mneumonics (i.e., integrating the letter shape into familiar objects, actions, and/or characters that reflect the letter sound; de Graaff et al., 2007; Shmidman & Ehri, 2010). Furthermore, studies have suggested including writing activities when teaching letters (e.g., Hall et al., 2015; Puranik et al., 2017). Thus, although research on the “science of alphabet instruction” has expanded (e.g., Roberts, 2021), we continue to have minimal understanding as to how to best support children’s alphabet learning (Piasta, 2023), especially for those at risk for reading difficulties who need effective, efficient, and evidence-based support.

### Uppercase facilitation effect in alphabet knowledge

One child factor that potentially influences children’s lowercase letter knowledge – and may have implications for effective alphabet instruction – is their knowledge of uppercase letters. Children tend to be more familiar with uppercase than lowercase letters (Evans et al., 2006; Turnbull et al., 2010), and knowing an uppercase letter positively predicts knowing the corresponding lowercase letter. This is termed the uppercase facilitation effect (e.g., Huang & Invernizzi, 2014; Pence-Turnbull et al., 2010; Treiman & Kessler, 2004). Pence-Turnbull et al. (2010), for example, used a cross-sectional design to examine whether children were more likely to know lowercase letter names if they knew the corresponding uppercase letter names. A total of 461 3- to 5- year-old children participated in the study, and the findings showed that children were 16 times more likely to know the name of a lowercase letter if they knew the corresponding uppercase letter than if they did not know the corresponding uppercase letter name. Notably, this effect also holds for children from economically disadvantaged backgrounds (Huang & Invernizzi, 2014), a group at higher risk for reading difficulties.

This uppercase facilitation effect may be due to the benefits of having one concept (i.e., knowledge of a letter name or letter sound) stored in multiple ways (i.e., two forms of the letter) and thus multiple ways to access the letter in long-term memory. Research to date has exclusively focused on the uppercase facilitation effect for letter name knowledge, not knowledge of letter sounds (e.g., Huang & Invernizzi, 2014; Justice et al., 2006; Pence-Turnbull et al., 2010; Treiman & Kessler, 2004). However, knowing letter names predicts knowing letter sounds, and many letter names include cues to letter sounds (Evans et al., 2006; Kim et al., 2010; Piasta & Wagner, 2010a), suggesting that the uppercase facilitation effect may also apply to letter sounds. This possibility is particularly relevant to children at risk for reading difficulties, who often struggle with the phonological aspect of letter-sound learning (Melby-Lervåg et al., 2012; Torppa et al., 2010).

Additionally, studies have shown that children are more likely to know letters that have visually similar shapes (Evans et al., 2006; Huang & Invernizzi, 2014; Pence-Turnbull et al., 2010; cf. Kim et al., 2020; Treiman & Kessler, 2004). For instance, Huang and Invernizzi (2014) examined whether knowledge of upper- and lowercase letter names was related in a sample of 5,020 kindergarten children from economically disadvantaged backgrounds, a population at elevated risk for reading difficulties, in the U.S. The findings showed that children were three times more likely to know lowercase letter names when the lowercase forms were visually similar to those of corresponding uppercase letters. Likewise, Vinter et al. (2023) evaluated the uppercase and lowercase letter-name knowledge of 122 3 to 5–6 years old French-speaking children. Findings showed that children’s lowercase letter-name knowledge was related to similarity in shape between lowercase and uppercase forms. Treiman and colleagues (2012, 2019) have also demonstrated the importance of visual similarity for children’s letter sound knowledge in large samples of English- and Hebrew-speaking children. Thus, given that many uppercase letters look similar to their corresponding lowercase letters, visual similarity might also account for children’s uppercase letter knowledge predicting their lowercase letter knowledge.

Notably, researchers studying the uppercase facilitation effect have used two analytic strategies in their work. Some used the sum scores of uppercase and lowercase letter naming to show associations between overall uppercase and lowercase letter knowledge (e.g., Treiman & Kessler, 2004; Vinter et al., 2023). Others used sophisticated analyses (e.g., logistic multilevel analysis) to investigate the uppercase facilitation effect at the individual letter level (Justice et al., 2006; Park & Piasta, 2023; Pence-Turnbull et al., 2010). Park & Piasta (2023), for example, measured the English letter-name knowledge of 56 U.S. emergent bilingual children. The findings showed that, similar to English monolingual children, emergent bilingual children also tended to know lowercase letter names when they already knew the corresponding uppercase letter name. This analysis strategy allows for a more rigorous test of the uppercase facilitation effect as it considers whether upper- and lowercase letter forms are known at the individual letter level and in the context of variation due to both differences across letters and differences across children (Kim et al., 2010; Piasta & Wagner, 2010b).

Despite rather consistent findings that children are more likely to know lowercase letter names when already familiar with their uppercase forms, a gap exists in our understanding of the uppercase facilitation effect. Specifically, we do not know if the effect applies to the learning of letter names or sounds, with corresponding instructional implications particularly for children at-risk of reading difficulties. Previous studies have been correlational and considered uppercase and lowercase letter-name knowledge concurrently (Huang & Invernizzi, 2014; Justice et al., 2006; Park & Piasta, 2023; Pence-Turnbull et al., 2010; Treiman & Kessler, 2004). The findings of available studies only speak to the fact that children are more likely to “know” a lowercase letter when they know the corresponding uppercase letter (i.e., are able to identify names for both cases at a given point in time), not whether they are more likely to *learn* lowercase letters if familiar with the uppercase form (i.e., whether children who are initially unable to identify lowercase letter names can do so at a later point in time). As such, we examined the uppercase facilitation effect in the context of learning over time. We hypothesized that children’s uppercase letter knowledge would facilitate their lowercase letter learning. Alternatively, the uppercase facilitation effect may reflect exposure or typical teaching patterns (e.g., teachers in the U.S. are more likely to teach uppercase letters first; Gerde et al., 2019), making this developmental pattern more a result of environmental factors rather than alphabet learning.

### The current study

Based on the available correlational evidence, scholars have recommended that early childhood teachers either teach uppercase letters first or teach both uppercase and lowercase letters simultaneously (Pence-Turnbull et al., 2010; Vinter et al., 2023), and teachers have reported enacting these recommendations (Gerde et al., 2019). To truly understand the uppercase facilitation effect and be able to make strong instructional recommendations, particularly for children at risk for reading difficulties, it is important to test the uppercase facilitation effect in the context of learning over time. It is also important to examine this using a within-subjects experimental design, which controls for environmental factors (e.g., other alphabet activities experienced at home or school). We addressed this gap by using a within-subjects design to examine whether knowing uppercase letters moderated the impact of alphabet instruction on lowercase letters. We considered letters as nested within each child using multilevel logistic regression.

## Method

### Participants

We analyzed data collected as a part of a larger U.S. alphabet intervention project which was approved by the University Internal Review Board. Data sharing was not part of the consent form signed by participants’ caregivers. One aim of the larger project was to compare the effects of multisensory instruction (MSI) versus visual-auditory instruction (VAI), and these findings are reported in Park et al (2025). A priori power analyses for addressing this aim indicated a sample size of 40 children to detect moderate effects. The analyses reported here addressed a separate aim specific to the uppercase letter facilitation effect.

To recruit participants, we collaborated with local early childhood programs to share study information, consent forms, and demographic surveys with caregivers of preschool-aged children. Additionally, we recruited via snowball sampling. Fifty-nine caregivers gave informed consent for their children’s participation. We applied several eligibility criteria: (a) caregiver consent, (b) age between 3 years, 5 months and 5 years, 5 months, (c) absence of severe disabilities, (d) ability to comprehend instructions and communicate in English, and (e) unfamiliarity with at least 12 lowercase letter names and sounds as assessed through production tasks. The latter signaled that these children might be at-risk for later reading difficulties due to low alphabet knowledge (i.e., know fewer than 18 uppercase and 15 lowercase letters; Piasta et al., 2012). We reviewed demographic surveys to verify if participants met criteria (a) through (c). For criterion (d), children with English as their primary language were included. For those children whose primary language was not English, we administered the Pre-Individual Proficiency Test (Pre-IPT; Stevens, 2010) to confirm that they had intermediate or advanced English oral proficiency levels and thus would be able to participate in lessons delivered in English. After confirming criteria (a) through (d) for 57 children, we evaluated their abilities to produce names and sounds for uppercase and lowercase letters to determine if they met criterion (e). We enrolled 42 eligible children in the study. Six children did not complete the study due to discontinuation of their childcare programs (*n* = 4) or chronic absenteeism (*n* = 2).

Of the 36 children who completed the study, 50% were girls, with an average age of 50 months at study start (*SD* = 7.12; range = 41 to 65). The racial composition was 42% Black, 22% White, and 25% Asian (11% unreported). Female caregivers’ highest education levels included high school diplomas (39%), Associate’s degrees (11%), Bachelor’s degrees (8%), and graduate degrees (9%); 20% did not have high school diplomas (13% unreported). Annual family incomes were below $25,000 for 47% of children, between $25,001 and $55,000 for 39%, between $55,001 and $75,000 for 3%, and above $75,001 for 9% (2% unreported). One child had an Individualized Education Plan. During the initial screening, children could name between 0 and 23 (*M* = 6.50, *SD* = 6.27) uppercase letters, and between 0 and 14 (*M* = 4.31, *SD* = 4.24) lowercase letters. For letter sound production, they could produce 0 to 5 uppercase letter sounds (*M* = 0.64, *SD* = 1.38) and 0 to 2 lowercase letter sounds (*M* = 0.28, *SD* = 0.57). Fourteen children used additional language(s) other than English at home, such that they were emergent bilinguals (EBs). EB children’s language backgrounds included Korean, Berber, Arabic, Spanish, and Nepali.

### Procedures

As a screening assessment, we evaluated children’s initial knowledge of letter names and sounds for all 26 English uppercase and lowercase letters. Among lowercase letters for which children could not produce either the letter name nor sound, we chose 12 for each individual child. We assigned each letter a difficulty value using those provided by Drouin et al. (2012). For each child, we rank ordered unknown letters from lowest to highest difficulty values and divided them into four sets of similar difficulty values. We randomly selected three letters from each set to obtain 12 letters in total. Then, we randomly assigned one letter per difficulty set to three groups: one group of four letters taught using MSI, one group of four letters taught with comparable VAI as explained below, and one group of four letters that were not taught, serving as controls. Given this procedure, the specific 12 letters taught to an individual child (e.g., e, n, p, k, u, r, t, z, y, w, a, x) differed from those taught to another individual child (e.g., n, d, v, x, y, w, f, s, u, i, a, k). Selected letters spanned the entire range of letter difficulty values. Per a one-way ANOVA, the letter difficulty values were similar across the three groups, *F*(2, 107) = 1.73, *p* =.182. As justified below, we collapsed across MSI and VAI letters to compare letters that were taught versus those that were not taught (control) for purposes of the current study. Per the within-subject experimental design, each child participated in all conditions, which controlled for non-study-related alphabet instruction and activities experienced at home, print environment, or in children’s preschool classrooms and also decreased other threats to internal validity.

All participating children received one-on-one lowercase alphabet instruction on MSI and VAI letters (see Park et al., 2025 for details of lessons). Research team members taught each child in a quiet location at their respective early childhood program or home. Uppercase letters were not explicitly taught during instruction, although lesson materials showed both uppercase and lowercase letter forms. We implemented three lessons per lowercase letter. Two review lessons were also provided for each subsequent letter selected for MSI and VAI instruction. We provided no instruction on control letters. Instruction for both MSI and VAI letters was systematic, explicit, and included elements recommended in available literature for teaching the alphabet. In a given lesson, children experienced multiple opportunities to see lowercase letters while saying letter names and sounds to support paired-associate learning (Roberts et al., 2019). All alphabet lessons were designed to last between 10 and 15 min and consisted of three parts: letter introduction, reading activity, and writing activity.

The difference between MSI and VAI instruction was that the letter introduction, reading activity, and writing activity in the MSI condition included tactile (e.g., tracing letters using sandpaper cards) and kinesthetic activities (e.g., tracing letters using sandpaper cards, enacting gestures and actions as mnemonic devices, writing letters using various multisensory materials, feeling vocal cords/breath/voicing when articulating sounds, observing/feeling mouth shapes when producing sounds) whereas the VAI condition included similar activities that avoided tactile and kinesthetic elements (e.g., tracing letters on plain paper, non- kinesthetic mnemonics, writing letters using regular paper and typical writing utensils, saying letter sounds without directing attention to articulation features or mouth shape). For the purpose of this study, we were interested in whether or not the letters were taught, as opposed to how letters were taught. Also, the intervention study showed no statistically significant differences between MSI and VAI conditions in letter learning (Park et al., 2025). As such, we combined the MSI and VAI intervention conditions into a single taught condition.

Finally, after completing alphabet lessons for both conditions (MSI and VAI), we administered posttest assessments on the selected 12 letters for each child. We then proceeded with planned analyses.

### Measures

We evaluated participating children’s letter name and sound production with the following tasks: (a) uppercase letter-name production, in which children were asked to name the displayed uppercase letter, (b) uppercase letter-sound production, in which children were asked to say the sound associated with the displayed uppercase letter, (c) lowercase letter-name production, in which children were asked to name the displayed lowercase letter, and (d) lowercase letter-sound production, in which children were asked to say the sound associated with the displayed lowercase letter. Scores from (c) and (d) served as independent variables. At screening, we assessed all 26 letters to ensure that children met eligibility criteria; at posttest, we only assessed the selected 12 letters for each child.

Assessors first presented 26 lowercase letters and then 26 uppercase letters to each child, with letters presented in random order within each task. For each letter, assessors asked the child for its name and corresponding sound. If a child gave a letter name instead of a sound or vice versa, they were prompted to provide the right type of response. For letters with multiple sounds, the sound taught during the alphabet lessons – which was the sound most commonly associated with the letter (e.g.,/g/for g) and lax vowel sounds – was considered correct, and children were prompted for an additional sound if they initially responded with a viable sound (e.g.,/j/for g, tense vowel sounds) other than the one taught during lessons. Previous research has successfully used similar tasks with children in this age range, reporting internal consistencies between 0.65 and 0.96 (Evans et al., 2006; Piasta et al., 2010). In this study, internal consistencies (Cronbach’s *α*) based on screening assessment of all 26 letters were 0.92 for uppercase letter naming, 0.86 for lowercase letter naming, 0.71 for uppercase letter sound production, and 0.16 for lowercase letter sound production. The low reliability for lowercase letter sound production is likely due to the low performance (i.e., floor effect) on the screening assessment, which was part of the eligibility criteria for this study (See also Piasta et al., 2022; Roberts et al., 2019). We were unable to estimate the internal consistencies of posttest assessments, as each participating child had different selected letters based on their unknown letters from the screening assessment. However, prior studies reported that the measures of internal consistencies maintained high levels when evaluating subsets of letters (Piasta et al., 2016; Roberts et al., 2019; Tortorelli et al., 2017).

## Results

The descriptive and correlation results among assessments are presented in Table 1. Descriptive statistics indicated that, at screening, children’s scores for the uppercase letter names corresponding to the 12 lowercase letters selected for each child ranged from 0 to 9 (*M* = 1.81, *SD* = 2.23). Scores for the corresponding 12 uppercase letter sounds ranged from 0 to 1 (*M* = 0.03, *SD* = 0.17). Scores for the 12 lowercase letter names and sounds were 0, per study eligibility criteria. Based on these descriptive statistics showing that children knew almost no uppercase or lowercase letter sounds at screening, we addressed the uppercase facilitation effects in the context of learning letter names only.

**Table 1:** Descriptives and correlations among screening and posttest measures

Measure	M	SD	Range	1	2	3	4	5	6	7	8
Uppercase											
1. T1 LN production	1.81	2.23	0–9	−	0.32	0.95^**^	0.28	0.68^**^	0.45^**^	0.52^**^	0.43^**^
2. T1 LS production	0.03	0.17	0–1		−	0.34^*^	0.87^**^	0.08	0.24	0.04	0.17
Lowercase											
3. T1 LN production	0	0	0			−	0.32	0.63^**^	0.37^*^	0.44^**^	0.35^*^
4. T1 LS production	0	0	0				−	0.06	0.27	0.04	0.24
Uppercase											
5. T2 LN production	5.69	4.13	0–12					−	0.61^**^	0.90^**^	0.65^**^
6. T2 LS production	2.53	2.60	0–10						−	0.60^**^	0.82^**^
Lowercase											
7. T2 LN production	4.67	3.21	4.67							−	0.74^**^
8. T2 LS production	2.47	2.55	2.47								−

Note. *N* = 36. T1 = Screening; T2 = posttest. LN = letter name; LS = letter sound

^*^p <.05. ^**^p <.01. ^***^p <.001

We analyzed the letter name data using multilevel logistic regression with letters (Level 1) nested within children (Level 2). The purpose of the analysis was to examine the likelihood of correctly naming a given lowercase letter at posttest, considering both whether the lowercase letter was taught and whether the child knew the corresponding uppercase form at the time of the screening. We used this analysis to determine whether uppercase letter knowledge moderated lowercase letter learning. A dummy-coded variable indicating whether the child responded correctly to each lowercase letter on the posttest served as the independent variable (1 = correct response, 0 = incorrect response); recall that, per eligibility and letter selection criteria, all children scored 0 for their 12 selected lowercase letters at screening, thus posttest scores alone represent learning. We included whether the child correctly responded to the uppercase letter during the screening (1 = correct response, 0 = incorrect response), whether the letter was taught (taught via either MSI or VAI = 1, control = 0), and the uppercase letter response × letter type interaction as Level-1 dependent variables. Results are in the metric of log-odds; a log-odds value of 1 indicates chance occurrence. In other words, children are equally likely to identify a letter correctly or incorrectly. When the log-odds value is above 1, an increase in the independent variable corresponds to children having a higher probability of accurately identifying a letter. Conversely, a log-odds value below 1 indicates that children have a lower likelihood of correctly naming a letter with an increase in the independent variable.

**Fig. 1:**
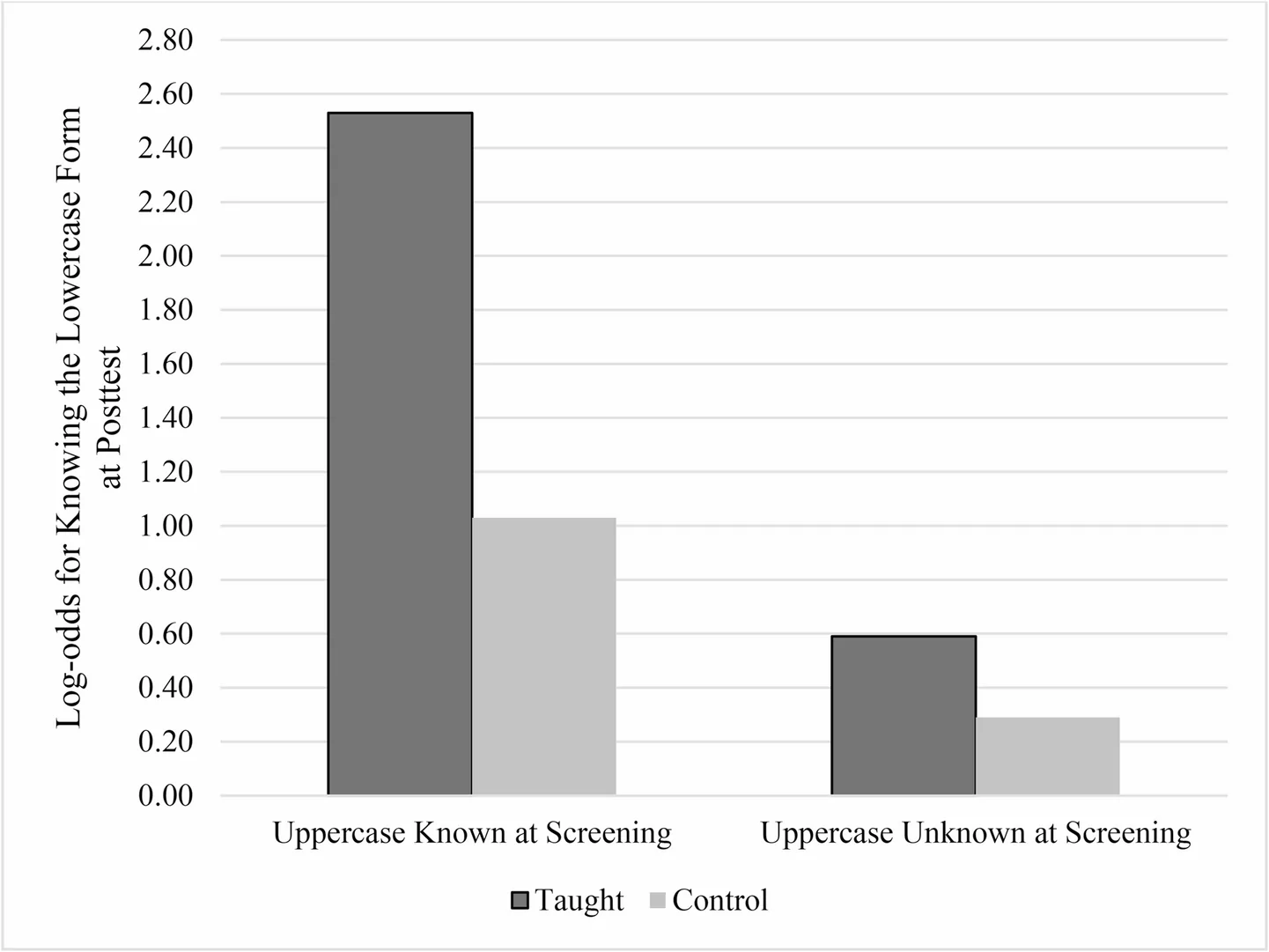
Log-odds of learning lowercase letter names

The results are presented in Table 2; Fig. 1. The log-odds was 0.29 when a lowercase letter was not taught and the child did not know the corresponding uppercase form. The log-odds was 1.03 when a lowercase letter was not taught and the child knew the corresponding uppercase form. When a lowercase letter was taught but the child did not know the corresponding uppercase form, the log-odds was 0.59. When a lowercase letter was taught and the child knew the corresponding uppercase form, the log-odds was 2.53. Despite the descriptive pattern, however, the interaction effect was not statistically significant, *β* = 0.18, *p* =.789. Thus, knowledge of the corresponding uppercase letter did not moderate effects of lowercase letter instruction.

**Table 2:** Examining uppercase facilitation effect using multilevel logistic regression model

	Lowercase Letter Name			
	Coeff (SE)	*p*	Odds ratio	CI
Intercept	−1.24 (0.29)	< 0.001	0.29	[0.16, 0.52]
Taught letter	0.72 (0.27)	0.009	2.05	[1.20, 3.51]
Uppercase letter known at screening	1.27 (0.54)	0.020	3.55	[1.22, 10.28]
Taught letter x Uppercase letter known at screening	0.18 (0.66)	0.789	1.19	[0.33, 4.36]

Note. *N* = 36. 12 selected letters were nested with each child. Coeff = coefficient. CI = confidence interval. Estimates were not calculated for the lowercase letter sound outcome because the children knew almost no uppercase letter sounds in the screening assessment

There were statistically significant main effects as to whether a letter was taught (*β* = 0.72, log odds = 0.59, *p* =.009) and whether children correctly responded to the uppercase letter during the screening (*β =* 1.27, log odds = 1.03, *p* =.020). These results indicate that children learned lowercase letter names when taught and also that children were more likely to know (but not necessarily learn) lowercase letter names when they already knew the name of the corresponding uppercase form. The children were 2.05 times more likely to know the name of a lowercase letter on the posttest if the lowercase letter was taught than if it was not taught. Additionally, children were 3.55 times more likely to know the name of a lowercase letter on the posttest if they knew the name of the corresponding uppercase letter on the screening assessment than if they did not.

### Post-hoc analyses

In addition to the a priori analyses described above, we also ran two post-hoc analyses. First, given that the number of uppercase letters known at the screening and posttest assessments were correlated [*r*(431) = 0.37, *p* <.001], we ran a post-hoc analysis using children’s knowledge of uppercase letters at posttest to see whether the findings paralleled the original analyses. Similarly to the original analysis, whether the child responded correctly to each lowercase letter on the posttest served as the outcome variable (1 = correct response, 0 = incorrect response). We included whether the child correctly responded to the uppercase letter at the posttest assessment (1 = correct response, 0 = incorrect response), whether the letter was taught (taught via MSI or VAI = 1, control = 0), and the uppercase letter response × letter type interaction as Level-1 predictors. The findings mirrored the original results. The interaction effect was of the same magnitude and not statistically significant, *β* = 0.18, *p* =.760. There was a statistically significant main effect for knowing the uppercase letter at posttest (*β =* 3.00, *p* <.001). There was no statistically significant main effect for whether a letter was taught (*β =* 0.33, *p* =.495).

Second, considering that the uppercase facilitation effect may be due to visual similarity between uppercase and lowercase letters (Evans et al., 2006; Huang & Invernizzi, 2014; Pence-Turnbull et al., 2010; Treiman & Kessler, 2004), we ran a sensitivity analysis including visual similarity between upper- and lowercase letters as a covariate. We obtained visual similarity values from Boles and Clifford (1989). They asked undergraduate students to rate the similarity of upper- and lowercase letters and used cluster analyses to determine similarity values for each uppercase-lowercase letter pair. We divided these values by 100 for the analysis (see Table 3) to put them on a similar scale as our other variables. We found the same pattern of results as the original findings. The interaction effect was not statistically significant, *β* = 0.18, *p* =.786. There were statistically significant main effects as to whether a letter was taught (*β* = 0.79, *p* =.005) and whether children correctly responded to the uppercase letter during the screening (*β =* 1.56, *p* =.006). There was also a statistically significant main effect for visual similarity (*β* = 0.44, *p* =.007).

**Table 3:** Visual similarity value between uppercase and lowercase letters (from Boles & Clifford, 1989)

Letter	Visual similarity value
A/a	2.37
B/b	2.63
C/c	4.13
D/d	2.42
E/e	2.59
F/f	3.59
G/g	2.21
H/h	3.21
I/i	3.13
J/j	3.79
K/k	4.34
L/l	2.55
M/m	3.34
N/n	3.04
O/o	4.34
P/p	4.13
Q/q	2.25
R/r	2.33
S/s	4.17
T/t	3.09
U/u	4.09
V/v	4.29
W/w	4.09
X/x	4.17
Y/y	3.09
Z/z	4.42

Note. Higher values indicate greater similarity between uppercase and lowercase forms for each letter

## Discussion

We examined whether the uppercase facilitation effect held in the context of alphabet learning while controlling for any between-child differences in additional alphabet activities conducted at home or school. Specifically, we analyzed whether knowing uppercase letter names moderated the impact of alphabet instruction on lowercase letter names for children with low alphabet knowledge and thus potentially at-risk for reading difficulties. The current study extended the prior literature by rigorously testing this effect within an experimental design and using analyses suitable to the design and data, with letters nested within children. We included not only monolingual participants but also those from diverse language backgrounds. As such, our sample reflects the range of children in U.S. classrooms who may experience reading difficulties. Our results indicated that children learned lowercase letter names when taught, yet, there was no uppercase facilitation effect evident in the context of learning. We also found that children were more likely to know (but not necessarily learn) lowercase letter names when they already knew the name of the corresponding uppercase letter. Below, we discuss our major findings along with implications, study limitations, and future directions.

First, we did not find that the uppercase facilitation effect applied to *learning* lowercase letters within our sample. Despite a descriptive pattern showing that lowercase letters were most likely to be learned when taught and the name of the uppercase form was known, there was no statistically significant interaction these variables. In other words, the uppercase facilitation effect was apparent for all selected letters for each child, even for control letters that were not taught. Moreover, the interaction effect was not of a magnitude typically considered practically significant (*d* = 0.10). The latter indicates that, even though we were likely underpowered to detect statistically significant moderation effects in this study (Sommet et al., 2023), any such effects would be small and likely not instructionally meaningful.

These results suggest that the uppercase facilitation effect may be a result of environmental factors rather than alphabet learning. Specifically, the uppercase facilitation effect may reflect exposure or typical teaching patterns, which would explain our similar effects (i.e., lack of moderation) for letters that were and were not taught. For example, parents and teachers may focus more on uppercase letters – especially children’s first initials – as opposed to lowercase letters (Dougherty Stahl, 2014; Gerde et al., 2019; Treiman & Broderick, 1998; Vander Hart et al., 2010). Moreover, our post-hoc analysis not only replicated the lack of interaction effect after accounting for visual similarity but also showed a main effect of the latter, with children more likely to know lowercase letters that were more visually similar to their uppercase forms. Thus, our findings do not rule out visual similarity as a plausible explanation for the uppercase facilitation effect (Huang & Invernizzi, 2014; Pence-Turnbull et al., 2010; Treiman & Kessler, 2004; Treiman et al., 2013).

Second, we did find a main effect of knowing the uppercase letter form: Children were 3.55 times more likely to know – but not necessarily learn – the name of a lowercase letter if they already knew the name of its uppercase form. This finding aligns with prior research showing that, for U.S. children, uppercase letter names are more likely to be known/less difficult than lowercase letter names (Anthony et al., 2021; Bowles et al., 2014; Drouin et al., 2012). This finding also replicates and extends the uppercase facilitation effect noted in previous literature (e.g., Evans et al., 2006; Pence-Turnbull et al., 2010; Treiman & Kessler, 2004). Specifically, we replicated the uppercase facilitation effect with children who had low alphabet knowledge and extended prior literature in which children’s alphabet knowledge was measured at a single time point (e.g., Huang & Invernizzi, 2014; Pence-Turnbull et al., 2010; Treiman & Kessler, 2004) to confirm that children who knew the name of an uppercase letter were more likely to know the name of the corresponding lowercase letter about 3 months later than if they did not know the name of the uppercase letter.

Notably, the magnitude of our effect indicating uppercase facilitation for knowing a lowercase letter is considerably smaller than those previously reported. Pence-Turnbull et al. (2010) found that preschool children were 16.09 times more likely to know a lowercase letter name when the uppercase form was known, and Park & Piasta (2023) found that preschool children who were emergent bilinguals were 10.06 times more likely to know a lowercase letter name when they already knew the uppercase form. The difference in magnitude may reflect the difference between knowing lowercase and uppercase letters concurrently versus longitudinally. This explanation is supported our finding of a larger effect (*β =* 3.00 versus 1.27) when considering uppercase letter name knowledge at posttest, measured concurrently with the lowercase letter name knowledge outcome. It may also reflect the fact that, in the current study, children received explicit alphabet instruction on some letters. Teachers of children participating in the Pence-Turnbull et al. (2010) study reported using Creative Curriculum (Dodge et al., 2002), High/Scope (Hohmann et al., 1995), or the Language-Focused Curriculum (Bunce, 1995) and, in the larger study from which the current sample was drawn, teachers primarily used Creative Curriculum or High/Scope. None of these curricula have consistently demonstrated impacts on children’s print knowledge, which includes alphabet knowledge, per the What Works Clearinghouse (see also Lonigan et al., 2011; Schachter et al., 2020). Finally, the difference in magnitude may be due to the nature of our sample, comprising children at-risk of later reading difficulties due to low alphabet knowledge.

Third, we found statistically significant – and large – main effects of alphabet instruction, despite no significant interaction effect. Children were 2.05 times more likely to know the name of a taught lowercase letter than an untaught lowercase letter. This translates to an approximate effect size of *d* = 0.40 (Huang & Invernizzi, 2014), which is considered large and educationally meaningful (Kraft, 2020). The main effect indicates that, regardless of whether children knew the uppercase form or not, teaching letters improved their lowercase letter name knowledge. This is not unexpected, given that the alphabet lessons used in the current study incorporated evidence-based instructional practices and had shown large effects on alphabet outcomes (*d* > 0.99 for letter name and sound production; Piasta et al., 2022). These results converge with past evidence in showing the positive effect of systematic and explicit alphabet instruction that leverages paired-associate learning (Piasta & Wagner, 2010b; Roberts et al., 2020). The findings also add to the emerging literature showing that such instruction is effective for children who are emergent bilinguals (Roberts et al., 2018, 2019) and for children who may be at risk for reading difficulties due to low alphabet knowledge (Park et al., 2025). Importantly, given the experimental designs of this and other studies, the effect of explicit alphabet instruction is above and beyond any alphabet instruction that children may have experienced in their educational programs or at home.

### Limitations and future directions

We note several limitations. First, on average, children knew the names of fewer than two of the uppercase letters individually selected for instruction at the time of screening. The limited variance in the predictor may have restricted any interaction effects, although we also did not find interaction effects when using the less-restricted posttest uppercase knowledge scores (average of six uppercase letter names known). The restricted variance is problematic because it reduces statistical power and attenuates observed relations. Future work would ideally include a sample demonstrating greater variability in their initial uppercase letter knowledge. Second, we were unable to test the uppercase facilitation effect for letter-sound knowledge because of the floor effect on this variable at the time of screening and posttest. To our knowledge, all work to date has examined the uppercase facilitation effect for letter-name knowledge only, not for letter-sound knowledge (e.g., Huang & Invernizzi, 2014; Pence-Turnbull et al., 2010). Future research is needed to understand whether the uppercase facilitation effect applies to children’s letter-sound learning. For example, subsequent studies could implement eligibility criteria that require children to demonstrate at least a basic level of uppercase letter-sound knowledge at screening while also knowing few lowercase letter sounds to reduce the likelihood of a floor effect. Understanding the extent to which uppercase facilitation applies to letter-sound learning is especially theoretically and practically important, given the current lack of evidence on this effect for letter-sound knowledge. This line of work is particularly critical for children at risk for reading difficulties, who often struggle with the phonological aspect of letter-sound learning (Melby-Lervåg et al., 2012; Torppa et al., 2010).

Third, the findings of the study cannot speak to situations in which both uppercase and lowercase letters are taught. Uppercase letters were not explicitly taught to children in the current study, although uppercase letters were depicted on the letter cards and in the picture books to which children were exposed during the alphabet instruction. Future studies may consider explicitly teaching uppercase letters as well to further investigate the uppercase facilitation effect in the context of learning. The current study design, teaching only lowercase letters, however, was the most suitable research design to address our research aim (i.e., examining whether “knowing” uppercase letters moderated the impact of alphabet instruction on lowercase letters).

## Conclusion

We found evidence of the uppercase facilitation effect, even for control letters that were not taught. The uppercase facilitation effect may therefore reflect exposure or typical teaching patterns rather than a learning effect. We also found that children—namely those at-risk for reading difficulties due to low alphabet knowledge—successfully learned lowercase letters when they received explicit instruction on them. Together, these findings do not support the notion that teaching uppercase letters is necessary to improve lowercase letter learning. Rather, children appear to learn lowercase letters when they are explicitly and systematically taught, regardless of uppercase knowledge. Thus, it is important that educators assess both uppercase and lowercase letter knowledge in their classrooms, with particular attention to learners in need of alphabet knowledge support, to identify specific gaps and provide targeted, explicit instruction on unknown letters. Curriculum developers and instructional leaders might also review pacing guides and materials to ensure that lowercase letter instruction is introduced early and taught systematically, rather than treated as a secondary goal after uppercase mastery. In this way, the present findings can inform ongoing efforts to align classroom practices, assessment, and curriculum decisions with research on effective alphabet instruction.

## Data Availability

No datasets were generated or analysed during the current study.
